# Effect of peripheral laser photocoagulation retinopexy on macular morphology and optic nerve fiber layer thickness; a prospective case series

**DOI:** 10.1186/s12886-022-02681-8

**Published:** 2022-11-25

**Authors:** Mohammadkarim Johari, Salman Khani, Abdulrahim Amini, Alireza Bolkheir

**Affiliations:** 1 Poostchi Ophthalmology Research Center, Department of Ophthalmology, School of Medicine, Shiraz University of Medical Sciences, Shiraz, Iran; 2 Hormozgan University of Medical Sciences, Bandar Abbas, Iran

**Keywords:** Peripheral laser photocoagulation retinopexy, Central foveal thickness, Retinal nerve fiber layer thickness, Epiretinal membrane

## Abstract

**Purpose:**

The goal of the research was to determine the incidence of microstructural alterations in the macula and optic nerve head (ONH) occurred in eyes treated with peripheral laser photocoagulation retinopexy.

**Methods:**

Patients with retinal breaks, retinal holes, retinal dialysis, and lattice degenerations who required peripheral laser photocoagulation retinopexy were recruited in this prospective case series investigation. We performed preoperative and postoperative evaluations, including best corrected visual acuity (BCVA), slit lamp examination, intraocular pressure (IOP) measurement, funduscopic examination, and macular and ONH optical coherence tomography (OCT).

**Results:**

Thirty-three eyes of the twenty-three patients enrolled in this study, 14 of which were female. The mean age of the sample was 45.12 ± 9.12 years. The mean refractive error was − 2.45 ± 1.12 Diopters (D). The most prevalent reason for peripheral laser photocoagulation retinopexy was retinal thinning with symptomatic lattice degeneration (90%), followed by retinal hole and break (7%) and retinal dialysis (3%). Between preoperative and postoperative (6-month) evaluation, there was no statistically significant difference in BCVA (*P* = 0.82), IOP (*P* = 0.54), central foveal thickness in macular OCT (*P* = 0.39), or global retinal nerve fiber layer (RNFL) thickness (*P* = 0.51). There was no association between changes in central foveal thickness and global RNFL thickness and total laser spot numbers (*r* = − 0.17 *P* = 0.21, *r* = 0.06 *P* = 0.60, respectively). None of patients developed cystoid macular edema (CME) or macular epiretinal membrane (ERM) .

**Conclusion:**

We found that OCT parameters were not significantly affected by laser retinopexy in patients with high-risk peripheral retinal lesions, also none of our patients had developed ERM, vitromacular traction or CME at 6 months follow up periods.

## Introduction

Posterior vitreous detachments are a frequent benign ocular condition caused by vitreous syneresis. The vitreous gradually liquefies (syneresis) over time, allowing the posterior hyaloid to separate from the macula and optic nerve. The vitreous then compresses anteriorly toward the vitreous base when this happens. The vitreous may exert enough strain on the retina during this anterior march to cause a retinal tear [1]. Around 5% of the adult population has symptomatic retinal tears [2]. According to several research, between 2 and 18% of retinal tears result in retinal detachment [3, 4]. Symptomatic retinal tears are associated with a much greater rate of retinal detachment than asymptomatic tears [4]. Laser retinopexy (laser photocoagulation to seal the tear) and cryoretinopexy (freezing treatment to seal the tear) are the two primary therapies for retinal breaks. The therapeutic technique to be followed should preferably be determined by the location and number of retinal breaks [1]. Numerous practitioners feel that cryoretinopexy increases the likelihood of developing an epiretinal membrane (ERM) and proliferative scarring. As a result, cryoretinopexy has lost popularity [1]. While sealing the retinal tear decreases the risk of progression to retinal detachment from 50 to 5%, it may result in the development of microstructural changes (such as ERM) in the macula [5–7]. The goal of this prospective research was to identify for the first time the occurrence of microstructural alterations in the macula and optic nerve head (ONH), such as ERM and cystoid macular edema (CME) development, after intensive peripheral laser photocoagulation retinopexy.

## Methods

This is case series research that is being conducted prospectively. Between January and June 2021, this study included all cases of retinal breaks, retinal holes, retinal dialysis, and lattice degenerations that required peripheral laser photocoagulation retinopexy at the ophthalmology clinics of Shiraz University of Medical Sciences (referral eye clinics in southern Iran). Prior to the trial, we notified eligible participants about the procedure and requested that they read and sign the written informed consent statement. The Shiraz University of Medical Sciences’ Ethics Committee accepted and approved the study’s protocol (code: IR.SUMS.MED.REC.1399.258). Participation was voluntary, and the study adhered to all of the Declaration of Helsinki’s standards for human subject’s research throughout. Patients with retinal dialysis, symptomatic or asymptomatic retinal tears or holes (single or multiple) with 1 clock hour of subretinal fluid, or those with symptomatic peripheral lattice degeneration with atrophic holes prolonged 6 clock hours met the inclusion criteria. The most common symptom in patients with peripheral lattice degeneration that needed peripheral laser retinopexy was flashing. All patients required continuous or intermittent peripheral laser photocoagulation retinopexy for ≥12 clock hours. Patients with myopia greater than 5 diopters, hyperopia greater than 3 diopters, pre-existing ERM, pre-existing vitromacular traction (VMT), macular hole, any type of macular dystrophy, diabetes and diabetic retinopathy, uveitis, retinal vascular occlusion, previous intraocular surgery (except cataract surgery within more than the previous 6 months), any type of glaucoma (as well as intraocular pressure (IOP) ≥21) and history of trauma were excluded. All patients had pre- and postoperative examinations and were handled by a single retina surgeon. Preoperative evaluation included best corrected visual acuity (BCVA), slit lamp examination, IOP measurement using Goldmann tonometry, description of the macula using a contact lens examination or a 90-diopter lens, and descriptions of the retinal breaks, retinal holes, retinal dialysis, and lattice degenerations, macular optical coherence tomography (OCT) and ONH OCT (Heidelberg-Germany). All participants involved in this study were followed for 6 months. At the last follow-up appointment, complete evaluation was done in the same manner as preoperative assessment. Finally, according to macular and ONH OCT, the incidence of ERM, VMT and CME formation were recorded. Mean central retinal thickness and peripapillary global retinal nerve fiber layer thickness changes to baseline were documented, also mean changes in BCVA and IOP from baseline were reported.

A 532 nm frequency-diode laser (Integre Pro ScanTM, Ellex medical corporation, USA) was used to treat the patient with a SuperQuad 160 contact lens (laser spot magnification of 2.0; Volk Optical Inc., Mentor, OH). Three to six rows of 500 𝜇 m spot size, 30 ms pulse length, and spot spacing of one width were employed to attach retina around the retinal lesions.

### Statistical analysis

The baseline characteristics of patients were described using descriptive statistics and expressed as mean (standard deviation, SD) for continuous data and frequency (%) for categorical data. To compare the mean OCT parameters and BCVA in eyes at baseline and at each follow-up visit, the chi-square and paired -tests were utilized. Kendall’s correlation coefficient was utilized to determine the relationship between OCT parameters and the total number of laser spots. For statistical purposes, BCVA was transformed to logarithm of the minimum angle of resolution (logMAR) units. SPSS version 18 was used for all analyses (SPSS Inc., Chicago, IL). A *P* < 0.05 was regarded significant.

## Results

Thirty-three eyes (23 patients) were enrolled with high-risk peripheral retinal lesions. The population’s mean age was 45.12 ± 9.12 years in this research. The research included nine men and fourteen women. Ten of the eyes were bilateral, five were right and eight were left. The mean refractive error was − 2.45 ± 1.12 Diopters (D). In these eyes, the most prevalent reason for peripheral laser photocoagulation retinopexy was retinal thinning with symptomatic lattice degeneration (90%), followed by retinal hole and break (7%) and retinal dialysis (3%). Table 1 summarizes the baseline demographics and clinical data of participants. In terms of the features of the peripheral laser photocoagulation retinopexy conducted, the average number of spots was 450 ± 210, with a minimum extension of 12 clock hours (23 eyes) and a maximum extension of 24 clock hours (ten eyes), as shown in Table 2. The mean BCVA before treatment was 0.22 ± 0.06 log units, which did not change significantly after 6 months (*P* = 0.82, Table 1). Pretreatment IOP was 17.51 ± 3.27 mmHg on average, with no significant change after 6 months (*P* = 0.54, Table 1). At baseline, the macular OCT characteristics were mostly normal; the mean pretreatment central foveal thickness was 276.78 ± 27.15 μm, which decreased to 274.30 ± 23.17 μm at 6 months following peripheral laser photocoagulation retinopexy. (*P* = 0.39). The mean global RNFL thickness before treatment was 98.27 ± 11.04 μm, increasing to 99.82 ± 10.59 μm after 6 months following peripheral laser photocoagulation retinopexy. (*P* = 0.51) Table 3 shows the changes in OCT parameters before and after laser retinopexy. There was no link between changes in central foveal thickness and total laser spot numbers, according to Kendall’s τ coefficient (*r* = − 0.17 *P* = 0.21). There was also no link between changes in total laser spot numbers and global RNFL thickness (*r* = 0.06 *P* = 0.60). None of our patients experienced laser induced macular edema, ERM, or VMT during a 6-month follow-up. There was no exudative or rhegmatogenous retinal detachment, choroidal detachment, or vitreous hemorrhage in any of the patients.

**Table 1:** Demographics and clinical findings of patients

Parameter	
Total eye number	33
Total patient number	23
Age (years; mean ± SD)	45.12 ± 9.12
Gender (no.; M(F)	9(14)
Laterality (no.; OU,OD,OS)	10,5,8
Average refractive error (diopters; mean ± SD)	−2.45 ± 1.12
Mean IOP before laser retinopexy (mmhg; mean ± SD)	17.51 ± 3.27
Mean IOP after laser retinopexy (mmhg; mean ± SD)	18.12 ± 2.55
Mean BCVA before laser retinopexy (logMAR; mean ± SD)	0.22 ± 0.06
Mean BCVA after laser retinopexy (logMAR; mean ± SD)	0.20 ± 0.06

*BCVA* best corrected visual acuity, *F* female, *M* male, *SD* standard deviation, *OU* both eyes, *OD* right eye, *OS* left eye, *IOP* intraocular pressure, *mmhg* millimeter of mercury, *log MAR* Log of Minimum Angle of Resolution

**Table 2:** Characteristics of laser parameters performed in our patients

Parameters	number	Mean ± SD
Laser spot size (μm)	33	588 ± 31
Laser power (mW)	33	323 ± 48
Total laser spot number	33	450 ± 210
Laser spot duration time(ms)	33	30
Laser wave length (nm)	33	532
Duration of treatment (min)	33	2 ± 1.21

*μm* micrometer, *mW* milliwatts, *ms* milliseconds, *nm* nanometer, *min* minute, *SD* standard deviation

**Table 3:** Changes in OCT parameters before and after peripheral laser photocoagulation retinopexy

Parameters	Before laser (mean ± SD)	After laser (mean ± SD)	*P* value
Centeral foveal thickness(μm)	276.78 ± 27.15	274.30 ± 23.17	0.39
Suprafoveal 3 mm thickness (μm)	340.27 ± 23.25	341.48 ± 14.58	0.73
Infrafoveal 3 mm thickness(μm)	339.75 ± 15.13	341.48 ± 13.23	0.25
Nasalfoveal 3 mm thickness(μm)	343.62 ± 17.86	345.58 ± 13.76	0.19
Temporalfoveal 3 mm thickness(μm)	330.21 ± 14.07	331.73 ± 13.70	0.24
Global RNFL thickness(μm)	98.27 ± 11.04	99.82 ± 10.59	0.51
Supraquadrant RNFL thickness(μm)	126.15 ± 23.58	124.21 ± 16.10	0.45
Infraquadrant RNFL thickness(μm)	123.54 ± 22.08	125.54 ± 23.91	0.58
Nasalquadrant RNFL thickness(μm)	73.75 ± 13.06	73.39 ± 14.15	0.75
Temporalquadrant RNFL thickness(μm)	79.12 ± 11.79	79.64 ± 79.64	0.84

*μm* micrometer, *RNFL* retinal nerve fiber layer, *OCT* optical coherence tomography, *SD* standard deviation

## Discussion

While laser retinopexy is typically a safe procedure, complications may arise. Inadvertent laser to the macula, choroidal effusions (especially when large amounts of laser are used), angle closure glaucoma, ERM formation, anterior segment laser burns, hemorrhage (of the retina, vitreous, or choroid), choroidal neovascular membrane formation, and the formation of new retinal breaks are just a few examples [8]. In this study, we used OCT to study the microstructural changes following laser retinopexy in patients with high-risk peripheral retinal lesions and found no statistically significant changes in central macular thickness and peripapillary global retinal nerve fiber layer thickness at 6 months follow up periods. Also, the incidence of ERM, VMT and CME following laser retinopexy in our patients were 0% at 6 months follow up periods.

Mester U, et al. studied retrospectively 2000 eyes with retinal breaks and degenerations that had been treated with argon laser photocoagulation, with follow-up ranging from 6 to 84 months (mean 46 months). Only petectal intra- and preretinal hemorrhages occurred after photocoagulation, which resolved after a few days. Complications after treatment were limited to ERM formation in the macular area in four eyes (0.2%). Three of these four eyes had much more applied laser energy (mean 34.4 mJ) than the other 2000 eyes (mean 7.2 mJ). They came to the conclusion that extensive photocoagulation is linked to an increased risk of ERM [9]. With the exception of one study that reported a significantly higher incidence of macular ERM formation, characterized as macular pucker, after laser treatment or cryoretinopexy (40 and 43%), previous studies showed a low (0 to 2.3%) incidence of macular ERM formation, after laser treatment or cryotherapy [6]. Two hundred five eyes with retinal tears treated with laser retinopexy, cryoretinopexy, or both were reviewed retrospectively by Saran BR and colleagues (with minimum 6 months follow-up). 10% of eyes treated with cryoretinopexy, 14% of eyes treated with laser, and 18% of eyes treated with both cryoretinopexy and laser retinopexy developed an ERM. There was no statistically significant difference in the incidence of macular ERM across the treatment modalities. They found that the difference in ERM incidence in their research compared to prior studies might be due to changes in categorization, detection sensitivity, and degree of treatment [6]. Blackorby BL,et al. evaluated retrospectively 2257 eyes with retinal tears treated with laser retinopexy, or cryoretinopexy. After treatment of retinal tears, 4.32% of cryoretinopexy eyes and 2.90% of laser retinopexy eyes had an ERM. The average time to ERM formation in their research was 11.5 months for cryoretinopexy and 12 months for laser retinopexy [1]. At the 6-month follow-up in our trial, none of the patients developed ERM. This outcome might be related to the fact that our research had fewer patients or a shorter follow-up period.

In patients treated with laser retinopexy, Khan Ashraf A and colleagues observed a 3.16% incidence of retinal detachment (RD). Early detachments (within 100 days of laser retinopexy) were caused by new or overlooked breaks in their study. In their investigation, the late RD was caused by the same treated retinal break. This finding might be owing to early undertreatment with sluggish advancement, or minor innocuous ocular trauma that progresses to RD via regions of weak retinal adhesion [10]. At the 6-month follow-up in our study, none of our patients developed RD.

Only a few studies have looked at changes in macular OCT following laser retinopexy. In one research, 25 myopic eyes with peripheral lattice degenerations had their macular OCT alterations assessed following laser photocoagulation. They found that following laser photocoagulation, the vitreoretinal tractions were blunted in macular OCT [11]. Ours is the first prospective research to show changes in macular and RNFL OCT following peripheral laser photocoagulation retinopexy (for retinal breaks, retinal holes, retinal dialysis, and lattice degenerations). After peripheral laser photocoagulation retinopexy, no statistically significant differences in macular and RNFL OCT values were identified.

There is no research on IOP variations following laser retinopexy in the literature. However, there have been some reports of alterations in IOP after panretinal laser photocoagulation (PRP). Increases in IOP of more than 6 mmHg are prevalent after PRP, with 32% to 94% of patients seeing a rise in IOP of more than 6 mmHg [12, 13]. In a research by Blondeau et al., all occurrences of PRP-related ocular hypertension were diagnosed within 2 hours after laser therapy [13]. No patients in our research had a rise in IOP throughout their follow-up.

This is the first prospective research to look at vitreoretinal interface disorders development, microstructural alterations in the macular, and RNFL OCT in patients who had peripheral laser photocoagulation retinopexy. Nonetheless, there are several drawbacks to this research, the most important limitation of this study is small sample size. Retinal tears that require laser retinopexy are not as common as other retinal diseases that require photocoagulation laser therapy, so larger series would be difficult to obtain in a single institution. On the other hand, photocoagulation laser is a safe procedure, and even a study with larger sample size would not add information for decision taking, if the incidence of macular changes were low. The other limitations are absence of a control group and the use of a non-randomized technique to recruit patients from a restricted number of medical institutions, which raises the risk of selection bias. Furthermore, we followed up with the patients for roughly 6 months, and longer follow-up periods may reveal a larger complication rate. More prospective clinical studies with larger sample numbers and longer follow-ups are required to assess microstructural changes in the macula and ONH, as well as to determine the incidence of vitreoretinal interface disorders development and clinically significant macular pucker following laser retinopexy.

## Conclusion

In this study, we found that OCT parameters were not significantly affected by laser retinopexy in patients with high-risk peripheral retinal lesions, also none of our patients had developed ERM, VMT or CME at 6 months follow up periods.

## Data Availability

Relevant data will be available by corresponding author on reasonable request. The data are not publicly available due to their containing information that could compromise the privacy of research participants.
